# A Global and Sector-Based Comparison of OCT Angiography and Visual Field Defects in Glaucoma

**DOI:** 10.1155/2022/6182592

**Published:** 2022-05-11

**Authors:** Alan W. Kong, Marcus L. Turner, Murtaza Saifee, Mohit Jethi, Marta Mora, Yvonne Ou

**Affiliations:** Department of Ophthalmology, UCSF School of Medicine, San Francisco, CA, USA

## Abstract

**Purpose:**

To evaluate the correlation of optical coherence tomography angiography (OCTA) and spectral-domain optical coherence tomography (SD-OCT) with visual field for global and sector-based indices among glaucoma and glaucoma-suspected eyes. *Patients and Methods*. This is a retrospective study, and in total, 48 glaucoma eyes and 31 glaucoma suspect eyes were included. The correlation between visual field parameters and radial peripapillary capillary (RPC) vessel density via OCTA was compared to the correlation with retinal nerve fiber layer (RNFL) thickness via SD-OCT. The RPC vessel density and RNFL thickness were divided into eight sectors, which included the temporal upper, temporal lower, superotemporal, inferotemporal, superonasal, inferonasal, nasal upper, and nasal lower sectors. Pearson correlations with 95% confidence intervals were calculated with resampling, and correlations were compared with a Fisher *Z* transformation.

**Results:**

Both RPC vessel density (*R* = 0.63, 95% CI [0.24, 0.86]) and RNFL thickness (*R* = 0.49, 95% CI [0.23, 0.69]) were correlated with the mean deviation when comparing global indices of glaucoma patients. In glaucoma suspects, the correlations between the mean deviation and RPC vessel density (*R* = 0.21, 95% CI [−0.05, 0.49]) and RNFL thickness (*R* = 0.01, 95% CI [−0.35, 0.39]) were not significant. Glaucoma eyes had the highest correlation between the mean sensitivity and RPC vessel density and RNFL thickness for the superotemporal, superonasal, temporal upper, and inferotemporal sectors.

**Conclusion:**

Across a diverse population and heterogeneous glaucoma types, RPC vessel density measurements correlate with global and sector-wise visual field indices similar to RNFL thickness.

## 1. Introduction

Optical coherence tomography angiography (OCTA) is a noninvasive imaging modality that provides qualitative and quantitative information on the optic nerve head (ONH) vascular network [1, 2]. There is an increasing body of evidence demonstrating that optic nerve blood flow impairment has a role in the pathogenesis of glaucoma [3]. These findings have been supported by structural imaging, indicating that decreased vessel density may be more significantly associated with severity of visual field (VF) damage independent of the structural loss measured by retinal nerve fiber layer (RNFL) thickness [4, 5]. However, there is conflicting evidence as to whether OCTA vessel density is superior to spectral-domain optical coherence tomography (SD-OCT)-measured RNFL thickness, as some studies have shown comparable diagnostic accuracy between OCTA and SD-OCT for differentiating healthy and glaucoma eyes [5].

Certainly, there is still an ongoing debate about whether OCTA contributes additional information to SD-OCT that can fortify the structure-function relationship in the pathophysiology of glaucoma. For instance, OCTA may be capable of detecting changes in the retinal microvasculature before damage is detectable on visual fields [6, 7]. OCTA has also been found to have high repeatability and reproducibility, have good discriminatory power between normal and glaucomatous eyes, and reach a floor effect at a more advanced disease stage than conventional SD-OCT [8–10]. Furthermore, OCTA may detect progression in glaucoma eyes to a degree that could enhance the understanding of the pathophysiological role of the blood flow in glaucoma [5, 11].

Recently, there have been advances on discovering region-specific vessel density correlations using Garway–Heath mapping that reflect the location-based visual field examination results. These results show that the superotemporal, superonasal, and inferotemporal sector changes of the ONH have the highest correlation with visual field pattern deviation changes [11–14]. However, many studies designed to evaluate OCTA only include subjects with primary open angle glaucoma (POAG) and normal tension glaucoma (NTG) [5–7,10–12, 14, 15]. Furthermore, some of these previous studies only measured differences between the people of European and African descent [5–7, 15] or studied a racially or ethnically homogeneous population [10–14].

The purpose of this study is to evaluate the correlation of OCTA and SD-OCT with visual field parameters to assess anatomical correlations of OCTA compared to VF in a heterogeneous group of glaucoma types, as well as in a racially/ethnically diverse population. In our study, we compared OCTA-measured radial peripapillary capillary (RPC) vessel density and SD-OCT-measured RNFL thickness with global visual field indices. We also performed a sector wise analysis that assessed the correlation of RPC vessel density or RNFL thickness with the corresponding visual field changes based on Garway–Heath mapping.

## 2. Methods

This was a retrospective study of patients who underwent OCTA during routine clinic visits from March 2017 to September 2018 at the Glaucoma Division, Department of Ophthalmology at the University of California, San Francisco. The study was approved by the Institutional Review Board of the University of California, San Francisco, and was conducted in accordance with the Declaration of Helsinki for research involving human subjects.

### 2.1. Subjects

Inclusion criteria included patients older than 18 years of age, a best-corrected visual acuity of 20/60 or better, and a spherical equivalent refraction within ±6.0 diopters. This patient population was heterogeneous and composed of subjects with different severities of disease and glaucoma types, including subjects diagnosed with primary open angle glaucoma, primary angle closure glaucoma, and pseudoexfoliation glaucoma. A diagnosis of glaucoma was defined based on evidence of optic nerve damage by either optic disc or RNFL structural abnormalities or reliable and reproducible visual field abnormality consistent with RNFL damage. Visual field defects included persistent scotoma on at least two consecutive standard automated perimetry tests. Abnormal disc appearance included neuroretinal rim thinning, localized or diffuse retinal nerve fiber layer defects, disc hemorrhages, or progressive narrowing of the neuroretinal rim with increased cupping, observed with slit-lamp biomicroscopy and a handheld lens or with SD-OCT imaging.

Glaucoma suspects were defined as patients with ocular hypertension, defined as having consistently elevated intraocular pressure (IOP) >21 mmHg or a suspicious optic nerve/RNFL in one or both eyes without visual field defects. Primary angle closure suspects were defined as having >180 degrees of the posterior pigmented trabecular meshwork not visible on static gonioscopy but without elevated IOP or optic neuropathy. Patients were excluded if they had evidence of other underlying retinal disorders.

### 2.2. Standard Automated Perimetry

Standard automated perimetry visual field tests were performed using Swedish Interactive Threshold Algorithm standard 24-2 threshold test (Humphrey Field Analyzer; Carl Zeiss Meditec, Inc, Dublin, CA). Participants with reliable tests defined as having less than 15% false-positive errors were included in the study. Visual fields that were found to have the following artifacts also were excluded: evidence of rim and eyelid artifacts, inattention or fatigue effects, or VF damage caused by a disease other than glaucoma.

Global indices such as the mean deviation (MD) and pattern standard deviation (PSD) were recorded. For specific sector analyses, pointwise total deviation (TDV), PSD, and mean sensitivity (MS) values were extracted using a validated, open-sourced script (https://pypi.org/project/hvf-extraction-script/) [16]. The script was used to extract pointwise data from the Ophthalmic Perimetry Values (OPV) DICOM files obtained directly from the Humphrey Visual Field device and PACS system. The sector-wise values were then calculated by averaging the pointwise values across each sector based on the Garway–Heath map [17].

### 2.3. OCTA Acquisition

Subjects underwent OCTA imaging (AngioVue, Optovue Inc., Fremont, CA, software versions 2016.2.0.35, 2017.1.0.151, and 2017.1.0.155). The AngioVue OCTA software quantifies the vessel density as the ratio of the area occupied by vessels (as measured by signal intensity) divided by the total measured area.

The peripapillary vessel density was derived from the images acquired with a 4.5 × 4.5 mm^2^ field of view centered on the optic disc similar to previous studies [5–7, 15]. The vessel density within the RNFL was measured from the internal limiting membrane (ILM) to RNFL posterior boundary. The RPC vessel density was also divided into eight sectors with AngioVue software, which included the temporal upper (TU), temporal lower (TL), superotemporal (ST), inferotemporal (IT), superonasal (SN), inferonasal (IN), nasal upper (NU), and nasal lower (NL) sectors. These corresponded to the visual field sectors numbered from 1 to 8, respectively.

Image quality was assessed for all OCTA scans. Images were excluded for poor-quality images with a signal strength index (SSI) less than 40. Images with poor clarity such as having a blurred image, with residual motion artifacts such as an irregular vascular pattern or a disc boundary on the en face angiogram, and/or with a local weak signal caused by floaters and RNFL segmentation errors were also excluded. This assessment was based on criteria set by previous studies [5–7, 15].

### 2.4. Spectral-Domain OCT Acquisition

SD-OCT (Avanti, Optovue Inc., Fremont, CA, software versions 2016.2.0.35, 2017.1.0.151, and 2017.1.0.155) uses a light source with a center wavelength of 840 nm and an A-scan rate of 70 kHz. The optic nerve head map image acquisition protocol was used to measure the circumpapillary RNFL thickness in a 10 pixel-wide band along a 3.45 mm diameter circle centered on the ONH [5–7, 15]. This was divided into the same eight sectors similar to the RPC vessel density.

### 2.5. Statistical Methods

Group characteristics were analyzed using Student's *t*-test for continuous variables and Fisher's exact test for categorical variables. To evaluate the relationship between the vessel density and VF parameters for global indices as well as for each corresponding sector, we calculated the Pearson correlation coefficients along with percentile-based 95% confidence intervals using a nonparametric bootstrap that was resampled at the individual level with replacement (1,000 iterations) to account for multiple eyes per person. Sectors were not compared against each other. Sector-wise VF parameters included average MS, TDV, and PSD in both decibels (dB) and 1/Lambert. This calculation was repeated for comparing the RNFL thickness and VF parameters. The correlation coefficients calculated from the previously mentioned parameters were compared using Fisher *Z* transformation. Finally, a linear regression model with robust standard errors was used to compare the visual field and structural indices to adjust for age and sex, and the model was clustered around the individual to account for multiple eyes per person. A *P* value < 0.05 was considered statistically significant. Data analyses were performed with R version 4.0.4 (R Foundation for Statistical Computing, Vienna, Austria).

## 3. Results

In this study, 66 individuals accounting for 114 eyes met our inclusion criteria and were evaluated for inclusion in the study. After screening for the scan quality and motion artifact, we had 50 individuals accounting for 77 eyes that were included for analysis. Of the 37 excluded eyes, 5 were excluded for having a signal strength index of less than 40, while the other eyes were excluded for either a motion artifact, irregular vascular patterns, or a floater artifact. In total, 48 glaucoma eyes (73.3 ± 12.3 years, average SSI 57.4 ± 7.7) and 29 glaucoma suspect eyes (64.9 ± 13.2 years, average SSI 62.7 ± 9.3) were included in the study. Demographic and ocular characteristics are described in Table 1. In addition, of the 48 glaucoma eyes, 75% had primary open angle glaucoma, 14.6% had primary angle closure glaucoma, and 10.4% had pseudoexfoliation glaucoma. Of the glaucoma suspect group, 65.5% were primary open angle glaucoma suspects, 20.7% were primary angle closure suspects, and 13.8% had ocular hypertension.

**Figure 1:**
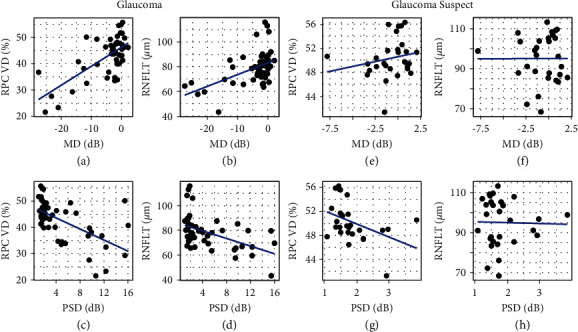
Scatterplots demonstrating linear relationship of structural and functional measures. Structural measures included radial peripapillary capillary (RPC) vessel density (VD) measured by optical coherence tomography angiography and retinal nerve fiber layer thickness (RNFLT) measured with spectral-domain optical coherence tomography. Functional measures from visual field testing include the mean deviation (MD) and pattern standard deviation (PSD). (a–d) Glaucoma subjects and (e–g) glaucoma suspects.

When comparing global indices, the mean deviation in glaucoma subjects correlated with the RPC vessel density (Figure 1(a)) with a Pearson correlation coefficient of 0.63 (95% CI [0.24, 0.86]). The mean deviation also correlated with the RNFL thickness (Figure 1(b)) with a correlation coefficient of 0.49 (95% CI [0.23, 0.69]). The difference between these two correlations was not significant (*P*=0.26). The pattern standard deviation in glaucoma subjects also correlated with the RPC vessel density with a correlation coefficient of −0.58 (95% CI [−0.82, −0.26], Figure 1(c)), and the correlation with RNFL thickness was −0.48 (95% CI [−0.70, −0.23], Figure 1(d)), which was also not statistically different (*P*=0.19). When adjusting for age and sex, the effect of the mean deviation on the RPC vessel density and RNFL thickness remained significant, while the effect of the pattern standard deviation was attenuated (Table 2). In glaucoma suspects, the correlation between the mean deviation and RPC vessel density or RNFL thickness was no longer significant with coefficients of 0.21 (95% CI [−0.05, 0.49]) and 0.01 (95% CI [−0.35, 0.39]), respectively (Figures 1(e) and 1(f)). However, the pattern standard deviation did significantly correlate with the RPC vessel density in glaucoma suspects with a coefficient of −0.43 (95% CI [−0.77, −0.10], Figure 1(g)), although this was no longer significant when controlling for age and sex (Table 3). The pattern standard deviation did not correlate with RNFL thickness with a coefficient of −0.03 (95% CI [−0.34, 0.24], Figure 1(h)). The correlations were similar when converting the mean deviation and pattern standard deviation into 1/Lambert (Supplemental Figure 1), which is consistent with previous studies that found converting decibels to 1/Lambert had comparable or weaker correlations [15,18,19].

**Figure 2:**
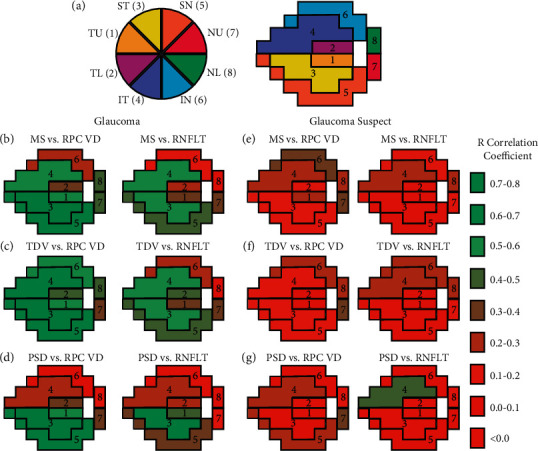
(a) Optic nerve head (ONH) sector divisions and the associated visual field sector numbering based on Garway–Heath mapping. These include the temporal upper (TU), temporal lower (TL), superotemporal (ST), inferotemporal (IT), superonasal (SN), inferonasal (IN), nasal upper (NU), and nasal lower (NL) sectors. Sector Pearson correlations shown for glaucoma and glaucoma suspect eyes between the mean sensitivity (MS, (b, e)), total deviation (TDV, (c, f)), and pattern standard deviation (PSD, (d, g)) with radial peripapillary capillary (RPC) vessel density (VD) or retinal nerve fiber layer thickness (RNFLT).

Sector naming is shown in Figure 2(a), and analysis for glaucoma subjects demonstrated that the correlations between the VF parameters (MS, TDV, and PSD) and RPC vessel density/RNFL thickness were best for the ST, SN, TU, and IT sectors (Figures *2(b)–*2(d), Supplemental Figures 2(a)–2(c), Table 4, and Supplemental Table 1). When controlling for age and sex, the mean sensitivity was a significant predictor for the RPC vessel density change for the TU, ST, IT, SN, and NL sectors (Supplemental Tables 2 and 3). Similar to the comparison of global indices, the visual field parameter correlations with the RPC vessel density were generally stronger than the correlations with the RNFL thickness, although none of these differences were significant. Glaucoma suspects showed weak correlations between the visual field parameters and structural measurements for all sectors (Figures 2(e)–2(g), Supplemental Figures 2(d)–2(f), Table 5, and Supplemental Table 4), even when controlling for age and sex (Supplemental Tables 5 and 6).

## 4. Discussion

Our study demonstrated that the RPC vessel density was found to have a stronger correlation with the MD than RNFL thickness, although the difference in this correlation was not statistically significant. Moreover, in glaucoma subjects, we show that the RPC vessel density significantly correlated with TDV, PSD, and MS, in particular in the ST, SN, and IT optic nerve head sectors, a relationship that has also been seen previously with the RNFL thickness [1, 10, 13, 20]. We also demonstrated that structural parameters from glaucoma suspects show a poor correlation with the corresponding visual field parameters.

We found similar vascular-anatomic change patterns present in glaucomatous eyes as seen in other studies on OCTA, corroborating data showing that ST and IT areas of the ONH are more vulnerable to nerve damage in patients with glaucoma [11–14]. Of note, some other studies have shown higher correlational values across sectors than what we demonstrated; however, most of these studies were analyzing one or two types of glaucoma and were mostly composed of homogeneous populations [7, 12, 21]. Future studies aimed at inspecting the utility of OCTA with more diverse populations should be implemented to validate a more universal utility for OCTA imaging in glaucoma. We believe our demonstration of a significant correlation between the OCTA vessel density and visual field parameters in this racially/ethnically diverse population further supports the clinical utility of OCTA.

A study that evaluated a nerve fiber trajectory-based method of anatomical correlation between OCTA and the VF yielded similar results to ours and those found in the literature [10]. This study used a structure-function analysis at the individual test points and then extrapolated nerve fiber trajectories through OCTA and VF parameters. Nevertheless, despite using a cluster-based analysis instead of a location-based one, our findings were consistent with this method, which illustrates how there may not be an advantage to using this location-based assessment. Moreover, while we did show that VF parameters had better correlations with the RPC vessel density over RNFL thickness, we did not find a significant difference between these two, which was seen previously in only the IT sector [12]. Other studies have shown that the vessel density may have similar to worse diagnostic capabilities compared to RNFL thickness for diagnosing open angle glaucoma when comparing the area under the receiver operating curve [21–24]. This suggests that the benefit of OCTA over SD-OCT may be marginal under certain parameters, and additional future studies that evaluate longitudinal data may better evaluate the utility of OCTA in glaucoma management.

While our study considered the peripapillary region for OCTA imaging, several studies have looked at the macula region for structure-function analysis [7, 22, 25, 26]. One study showed how macula VD-function analysis, which utilized Octopus perimetry to test the central 30-degree visual field, produces higher correlational values than the ST and IT peripapillary sectors [25]. This may indicate how there may be an improved structure-function relationship of the macula, possibly because there is an increased test-point density in the central macular area. The macula vessel density was not compared in this study, and future studies should further identify how the peripapillary capillary vessel density may be compared to macula data for OCTA imaging.

A strength of our study is our study population, which included a more heterogeneous set of glaucoma types and races/ethnicities. This suggests that the structure-function correlations noted in this study may be applicable to a broader group of patients than the previous studies with a more limited scope were able to demonstrate [5–7,10–15]. Furthermore, visual field parameters were extracted using a validated, open-sourced script [16], illustrating how this script can be applied for future structure-function studies.

One limitation of our study is that of the 116 eyes that met our clinical inclusion criteria, only 77 eyes met our imaging inclusion criteria due to poor signal strength index and motion artifacts. While the RPC vessel density does provide insight into the vascular network of glaucomatous and nonglaucomatous eyes through a fast and objective test, advances in the current image quality are needed to utilize this technology more broadly. Due to the nature of OCTA imaging and its software, there can be doubling of vessels, stretching defects, loss of detail, and line artifacts. Furthermore, while axial motion can be compensated for, the transverse motion from fixation changes still causes a majority of artifacts in OCTA [27]. While apparent artifacts were excluded, it is possible that less detectable artifacts could have a partial confounding effect on our results. In addition, we did not identify a correlation between the VF and structural parameters in glaucoma suspects. One possible explanation for this is that we grouped both open angle and angle closure suspects together, which may have different pathophysiology regarding changes in the blood flow at the optic nerve head [26].

In conclusion, we compared OCTA and OCT to VF parameters via a global and sector-based analysis and demonstrated that OCTA may play a beneficial role in glaucoma characterization. Our study highlighted how the RPC vessel density may offer a similar value in correlating with functional deficits in glaucoma compared to the RNFL thickness. For future clinical and diagnostic purposes, additional longitudinal studies are needed to be able to demonstrate whether OCTA can better evaluate the topographic and temporal changes in glaucoma and monitor disease progression. Using a racially diverse and heterogeneous glaucoma population, our study contributes to the field by further adding to the validity of OCTA as an objective and a structurally based method that may complement SD-OCT in order to assess glaucoma.

## Figures and Tables

**Table 1:** Demographics and ocular characteristics.

	Glaucoma (*N* = 48)	Glaucoma suspect (*N* = 29)	*P* value
Age (years)	73.3 ± 12.3	64.9 ± 13.2	0.01
BCVA (LogMAR)	0.12 ± 0.16	0.09 ± 0.11	0.35
IOP (mmHg)	13.4 ± 3.3	15.2 ± 3.3	0.03
Refractive error (SE; diopter)	−1.45 ± 2.73	−0.53 ± 1.68	0.07
Pseudophakic status (%)	56.3	27.6	0.02
Gender (% female)	70.8	79.3	0.61
Race (%)			0.96
White, non-Hispanic	54.2	51.7	
White, Hispanic	4.0	3.5	
Asian	31.3	31.0	
African American	4.2	3.5	
Other	6.3	10.3	
Hypertension (%)	45.8	48.3	1.00
Diabetes mellitus (%)	12.5	27.6	0.13
Mean deviation (dB)	−4.94 ± 7.19	−0.70 ± 2.03	<0.001
Pattern standard deviation (dB)	4.88 ± 4.34	1.82 ± 0.61	<0.001
Average RNFL thickness (*μ*m)	77.3 ± 12.0	95.1 ± 12.0	<0.001
Average GCC (*μ*m)	81.9 ± 12.2	93.5 ± 6.8	<0.001
Average vessel density (%)	42.3 ± 7.4	50.3 ± 3.3	<0.001
OCTA average SSI	57.4 ± 7.7	62.7 ± 9.3	0.01

BCVA: best corrected visual acuity, SE: spherical equivalent, IOP: intraocular pressure, RNFL: retinal nerve fiber layer, GCC: ganglion cell complex, OCTA: optical coherence tomography angiography, and SSI: signal strength index.

**Table 2:** Linear regression model with robust standard errors of visual and structural parameters adjusted for age and sex in glaucoma subjects.

*Linear regression estimates using mean deviation*			
Variable	RPC (vessel density, %)	Standard error	*P* value
Mean deviation (dB)	0.64	0.17	0.011
Age (years)	−0.18	0.10	0.118
Male sex	−0.72	2.10	0.737
Variable	RNFL (*μ*m)	Standard error	*P* value
Mean deviation (dB)	0.85	0.16	0.003
Age (years)	−0.07	0.22	0.75
Male sex	−2.94	4.18	0.49

*Linear regression estimates using pattern standard deviation*			
Variable	RPC (vessel density, %)	Standard error	*P* value

Pattern standard deviation (dB)	−0.89	0.44	0.087
Age (years)	−0.15	0.10	0.146
Male sex	1.16	2.30	0.619
Variable	RNFL (*μ*m)	Standard error	*P* value
Pattern standard deviation (dB)	−1.33	0.56	0.053
Age (years)	−0.04	0.20	0.863
Male sex	−0.409	4.35	0.926

**Table 3:** Linear regression model with robust standard errors of visual and structural parameters adjusted for age and sex in glaucoma suspect subjects.

*Comparisons using mean deviation*			
Variable	RPC (vessel density, %)	Standard error	*P* value
Mean deviation (dB)	0.21	0.23	0.405
Age (years)	−0.14	0.05	0.036
Male sex	0.71	2.35	0.775
Variable	RNFL (*μ*m)	Standard error	*P* value
Mean deviation (dB)	0.19	1.04	0.860
Age (years)	−0.06	0.26	0.832
Male sex	9.13	5.98	0.184

*Comparison using pattern standard deviation*			
Variable	RPC (vessel density, %)	Standard error	*P* value

Pattern standard deviation (dB)	−2.16	1.18	0.145
Age (years)	−0.12	0.043	0.028
Male sex	1.78	.97	0.413
Variable	RNFL (*μ*m)	Standard error	*P* value
Pattern standard deviation (dB)	−2.87	3.21	0.425
Age (years)	−0.03	0.28	0.914
Male sex	10.62	5.44	0.117

**Table 4:** Correlations of the RPC vessel density and RNFL thickness with the mean sensitivity, TDV, and PSD in the corresponding optic nerve head sector for glaucoma subjects.

Optic nerve head sector	R Pearson correlation with mean sensitivity (dB)		R Pearson correlation with TDV (dB)		R Pearson correlation with PSD (dB)	
	RPC vessel density (95% CI)	RNFL thickness (95% CI)	RPC vessel density (95% CI)	RNFL thickness (95% CI)	RPC vessel density (95% CI)	RNFL thickness (95% CI)
Temporal upper	0.56 (0.11, 0.77)	0.34 (−0.19, 0.71)	0.54 (0.05, 0.76)	0.36 (−0.12, 0.70)	0.52 (0.02, 0.83)	0.50 (0.17, 0.80)
Temporal lower	0.39 (0.09, 0.66)	0.22 (−0.04, 0.48)	0.43 (−0.02, 0.82)	0.43 (0.10, 0.67)	0.34 (0.05, 0.62)	0.27 (0.07, 0.47)
Superotemporal	0.79 (0.62, 0.89)	0.59 (0.35, 0.75)	0.79 (0.63, 0.90)	0.56 (0.34, 0.72)	0.80 (0.66, 0.90)	0.64 (0.40, 0.80)
Inferotemporal	0.52 (0.28, 0.73)	0.51 (0.28, 0.64)	0.63 (0.31, 0.86)	0.52 (0.33, 0.66)	0.25 (0.02, 0.54)	0.30 (0.16, 0.44)
Superonasal	0.69 (0.47, 0.84)	0.44 (0.11, 0.71)	0.66 (0.45, 0.82)	0.41 (0.10, 0.68)	0.68 (0.48, 0.85)	0.35 (-0.09, 0.71)
Inferonasal	0.24 (−0.11, 0.54)	0.14 (−0.29, 0.54)	0.53 (-0.08, 0.79)	0.30 (−0.17, 0.59)	0.08 (−0.30, 0.52)	−0.04 (−0.44, 0.45)
Nasal upper	0.42 (0.12, 0.62)	0.31 (−0.24, 0.67)	0.38 (0.08, 0.61)	0.31 (−0.26, 0.68)	0.37 (−0.08, 0.62)	0.15 (−0.49, 0.63)
Nasal lower	0.45 (0.15, 0.67)	0.08 (−0.24, 0.40)	0.42 (0.12, 0.63)	0.09 (−0.22, 0.43)	0.20 (−0.04, 0.41)	0.09 (−0.25, 0.43)

**Table 5:** Correlations of the RPC vessel density and RNFL thickness with the mean sensitivity, TDV, and PSD in the corresponding optic nerve head sector for glaucoma suspect subjects.

Optic nerve head sector	R Pearson correlation with mean sensitivity (dB)		R Pearson correlation with TDV (dB)		R Pearson correlation with PSD (dB)	
	RPC vessel density (95% CI)	RNFL thickness (95% CI)	RPC vessel density (95% CI)	RNFL thickness (95% CI)	RPC vessel density (95% CI)	RNFL thickness (95% CI)
Temporal upper	0.23 (−0.14, 0.53)	−0.15 (−0.59, 0.38)	0.04 (−0.32, 0.31)	−0.22 (−0.57, 0.23)	−0.20 (−0.44, 0.01)	−0.41 (−0.63, −0.14)
Temporal lower	0.20 (−0.33, 0.60)	−0.00 (−0.40, 0.35)	0.04 (−0.38, 0.44)	−0.02 (−0.43, 0.37)	−0.20 (−0.53, 0.12)	−0.31 (−0.67, 0.21)
Superotemporal	0.19 (−0.10, 0.45)	−0.11 (−0.42, 0.30)	0.11 (−0.24, 0.40)	−0.13 (−0.42, 0.28)	0.10 (−0.26, 0.41)	−0.06 (−0.32, 0.33)
Inferotemporal	0.29 (−0.02, 0.56)	0.28 (−0.19, 0.65)	0.16 (−0.10, 0.48)	0.24 (−0.20, 0.58)	0.27 (0.05, 0.57)	0.46 (−0.11, 0.46)
Superonasal	0.16 (−0.10, 0.44)	−0.03 (−0.42, 0.38)	0.17 (−0.04, 0.43)	−0.04 (-0.39, 0.31)	0.25 (−0.03, 0.51)	0.08 (−0.28, 0.37)
Inferonasal	0.36 (0.02, 0.66)	0.18 (−0.20, 0.47)	0.21 (−0.18, 0.55)	0.23 (−0.08, 0.50)	0.09 (−0.24, 0.42)	0.07 (−0.22, 0.39)
Nasal upper	0.38 (−0.12, 0.74)	0.16 (−0.24, 0.52)	0.22 (−0.32, 0.63)	0.18 (−0.16, 0.47)	0.39 (−0.16, 0.77)	0.07 (−0.28, 0.39)
Nasal lower	0.28 (0.01, 0.54)	0.22 (−0.18, 0.56)	0.29 (−0.02, 0.56)	0.26 (−0.09, 0.55)	0.16 (−0.22, 0.49)	0.19 (−0.11, 0.46)

## Data Availability

The data that support the findings of this study are available from the corresponding author, YO, upon reasonable request.

## Supplementary Materials

Supplementary Materials Supplemental Figure 1: scatterplots demonstrating linear relationship of structural and functional measures, using 1/Lambert units for visual field measures. Structural measures included radial peripapillary capillary (RPC) vessel density (VD) measured by optical coherence tomography angiography and retinal nerve fiber layer thickness (RNFLT) measured with spectral-domain optical coherence tomography. Functional measures from visual field testing include the mean deviation (MD) and pattern standard deviation (PSD). (A–D) Glaucoma subjects and (E–G) glaucoma suspects. Supplemental Figure 2: sector Pearson correlations shown for glaucoma and glaucoma suspect eyes between the mean sensitivity (MS (B, C)), total deviation (TDV (D, E)), and pattern standard deviation (PSD (F, G)) using 1/Lambert units with radial peripapillary capillary (RPC) vessel density (VD) or retinal nerve fiber layer thickness (RNFLT). Sectors of the optic nerve head are defined as the temporal upper (TU), temporal lower (TL), superotemporal (ST), inferotemporal (IT), superonasal (SN), inferonasal (IN), nasal upper (NU), and nasal lower (NL) sectors. Supplemental tables include tables evaluating structure-function correlations using 1/Lambert units for visual field measures. This file also contains the linear regression estimates of the effect of visual field measures (dB and 1/Lambert) while adjusting for age and sex.
