# Supplementary material for: A Global and Sector-Based Comparison of OCT Angiography and Visual Field Defects in Glaucoma
Source: J Ophthalmol. 2022 May 11;2022:6182592. doi: 10.1155/2022/6182592 (PMC9124140; doi:10.1155/2022/6182592)
Supplement: Supplementary Materials — Supplemental Figure 1: scatterplots demonstrating linear relationship of structural and functional measures, using 1/Lambert units for visual field measures. Structural measures included radial peripapillary capillary (RPC) vessel density (VD) measured by optical coherence tomography angiography and retinal nerve fiber layer thickness (RNFLT) measured with spectral-domain optical coherence tomography. Functional measures from visual field testing include the mean deviation (MD) and pattern standard deviation (PSD). (A–D) Glaucoma subjects and (E–G) glaucoma suspects. Supplemental Figure 2: sector Pearson correlations shown for glaucoma and glaucoma suspect eyes between the mean sensitivity (MS (B, C)), total deviation (TDV (D, E)), and pattern standard deviation (PSD (F, G)) using 1/Lambert units with radial peripapillary capillary (RPC) vessel density (VD) or retinal nerve fiber layer thickness (RNFLT). Sectors of the optic nerve head are defined as the temporal upper (TU), temporal lower (TL), superotemporal (ST), inferotemporal (IT), superonasal (SN), inferonasal (IN), nasal upper (NU), and nasal lower (NL) sectors. Supplemental tables include tables evaluating structure-function correlations using 1/Lambert units for visual field measures. This file also contains the linear regression estimates of the effect of visual field measures (dB and 1/Lambert) while adjusting for age and sex. [file 6182592.f1.zip › 6182592.f1/Supplemental Tables.docx]

Supplemental Table 1: Correlations of RPC vessel density and RNFL thickness with mean sensitivity, TDV, and PSD in the corresponding optic nerve head sector for glaucoma subjects

| Optic Nerve Head sector | R Pearson correlation with mean sensitivity (1/Lambert) | | R Pearson correlation with TDV (1/Lambert) | | R Pearson correlation with PSD (1/Lambert) | |
| --- | --- | --- | --- | --- | --- | --- |
|  | RPC vessel density (95% CI) | RNFL thickness (95% CI) | RPC vessel density (95% CI) | RNFL thickness (95% CI) | RPC vessel density (95% CI) | RNFL thickness (95% CI) |
| Temporal Upper | 0.33 (0.00, 0.57) | 0.07 (-0.12, 0.60) | 0.28 (-0.15, 0.57) | 0.12 (-0.20, 0.43) | 0.32 (-0.10, 0.65) | 0.45 (0.16, 0.70) |
| Temporal Lower | 0.31 (0.07, 0.54) | 0.23 (-0.07, 0.51) | 0.35 (0.03, 0.61) | 0.38 (0.11, 0.61) | 0.27 (0.03, 0.49) | 0.32 (0.05, 0.53) |
| Superotemporal | 0.65 (0.43, 0.80) | 0.43 (0.21, 0.62) | 0.67 (0.45, 0.82) | 0.45 (0.19, 0.64) | 0.81 (0.68, 0.89) | 0.58 (0.32, 0.74) |
| Inferotemporal | 0.53 (0.37, 0.69) | 0.56 (0.36, 0.71) | 0.58 (0.34, 0.76) | 0.55 (0.34, 0.72) | 0.25 (-0.02, 0.58) | 0.29 (0.10, 0.47) |
| Superonasal | 0.52 (0.29, 0.70) | 0.26 (0.01, 0.46) | 0.51 (0.28, 0.67) | 0.25 (-0.10, 0.46) | 0.58 (0.35, 0.77) | 0.21 (-0.14, 0.53) |
| Inferonasal | 0.23 (-0.08, 0.52) | 0.26 (-0.12, 0.60) | 0.34 (-0.02, 0.59) | 0.33 (-0.00, 0.61) | 0.05 (-0.37, 0.43) | 0.10 (-0.28, 0.48) |
| Nasal Upper | 0.33 (0.07, 0.54) | 0.10 (-0.28, 0.44) | 0.26 (0.01, 0.50) | 0.09 (-0.30, 0.45) | 0.19 (-0.13, 0.48) | -0.02 (-0.42, 0.36) |
| Nasal Lower | 0.28 (0.05, 0.49) | 0.07 (-0.16, 0.33) | 0.28 (0.04, 0.50) | 0.15 (-0.11, 0.42) | 0.15 (-0.07, 0.39) | 0.13 (-0.17, 0.48) |

Supplemental Table 2: Effect of mean sensitivity measured in decibels on RPC vessel density or RNFL thickness adjusting for patient demographics in glaucoma subjects

| Optic Nerve Head sector | Estimate for RPC (Vessel Density %) | | | | | |
| --- | --- | --- | --- | --- | --- | --- |
|  | Mean Sensitivity (dB, SE) | P Value | Age (years, SE) | P Value | Male Gender (SE) | P Value |
| Temporal Upper | 1.05 (0.26) | **0.036** | -0.05 (0.10) | 0.594 | -1.87 (2.00) | 0.361 |
| Temporal Lower | 0.68 (0.49) | 0.213 | -0.13 (0.15) | 0.396 | -1.15 (2.81) | 0.689 |
| Superotemporal | 1.35 (0.20) | **0.001** | -0.05 (0.11) | 0.672 | 0.49 (2.70) | 0.858 |
| Inferotemporal | 0.95 (0.20) | **0.023** | -0.19 (0.09) | 0.074 | 1.69 (2.37) | 0.486 |
| Superonasal | 0.99 (0.10) | **< 0.001** | -0.07 (0.14) | 0.612 | -1.75 (2.60) | 0.510 |
| Inferonasal | 0.55 (0.36) | 0.185 | 0.00 (0.13) | 0.998 | -2.37 (2.48) | 0.353 |
| Nasal Upper | 0.62 (0.25) | 0.078 | -0.14 (0.10) | 0.222 | 1.26 (2.81) | 0.660 |
| Nasal Lower | 0.77 (0.23) | **0.009** | -0.16 (0.12) | 0.229 | -3.33 (2.66) | 0.227 |
|  | Estimate for RNFL (um) | | | | | |
| Temporal Upper | 1.93 (0.42) | **0.026** | 0.48 (0.24) | 0.076 | -7.11 (5.76) | 0.233 |
| Temporal Lower | 0.73 (0.36) | 0.088 | 0.04 (0.20) | 0.851 | -0.40 (4.25) | 0.926 |
| Superotemporal | 1.99 (0.63) | **0.026** | 0.13 (0.31) | 0.68 | -2.48 (6.21) | 0.694 |
| Inferotemporal | 1.92 (0.48) | **0.028** | -0.45 (0.34) | 0.225 | 1.97 (8.62) | 0.822 |
| Superonasal | 1.46 (0.60) | **0.047** | 0.01 (0.43) | 0.986 | -6.75 (8.20) | 0.423 |
| Inferonasal | 0.86 (0.99) | 0.424 | 0.04 (0.27) | 0.896 | -6.51 (6.53) | 0.334 |
| Nasal Upper | 1.37 (0.71) | 0.138 | -0.08 (0.24) | 0.743 | -7.32 (5.19) | 0.177 |
| Nasal Lower | 0.25 (0.54) | 0.657 | 0.03 (0.23) | 0.910 | 0.42 (4.88) | 0.932 |

Supplemental Table 3: Effect of mean sensitivity measured in 1/Lambert on RPC vessel density or RNFL thickness adjusting for patient demographics in glaucoma subjects

| Optic Nerve Head sector | Estimate for RPC (Vessel Density %) | | | | | |
| --- | --- | --- | --- | --- | --- | --- |
|  | Mean Sensitivity (1/L, SE) | P Value | Age (years, SE) | P Value | Male Gender (SE) | P Value |
| Temporal Upper | 4.2e-3 (3.0e-3) | 0.196 | -0.04 (0.12) | 0.733 | -0.32 (2.11) | 0.880 |
| Temporal Lower | 1.5e-3 (2.1e-3) | 0.484 | -0.17 (0.16) | 0.304 | -0.66 (2.77) | 0.815 |
| Superotemporal | 1.8e-2 (4.3e-3) | **0.001** | 0.07 (0.14) | 0.630 | -0.43 (3.29) | 0.897 |
| Inferotemporal | 1.1e-2 (4.3e-3) | **0.027** | -0.07 (0.13) | 0.602 | 1.64 (2.29) | 0.486 |
| Superonasal | 1.5e-2 (3.6e-3) | **0.003** | -0.04 (0.13) | 0.765 | -2.89 (3.12) | 0.370 |
| Inferonasal | 0.01 (0.01) | 0.135 | 0.04 (0.14) | 0.783 | -2.99 (2.48) | 0.249 |
| Nasal Upper | 4.3e-3 (3.7e-3) | 0.268 | -0.16 (0.10) | 0.163 | 1.66 (2.97) | 0.583 |
| Nasal Lower | 3.4e-3 (3.3e-3) | 0.332 | -0.21 (0.14) | 0.171 | -2.04 (3.03) | 0.511 |
|  | Estimate for RNFL (um) | | | | | |
| Temporal Upper | 7.2e-3 (4.5e-3) | 0.137 | 0.49 (0.27) | 0.113 | -4.07 (5.79) | 0.493 |
| Temporal Lower | 4.9e-3 (2.4e-3) | 0.064 | 0.11 (0.22) | 0.634 | -0.61 (4.23) | 0.888 |
| Superotemporal | 0.02 (0.01) | **0.017** | 0.25 (0.38) | 0.521 | -3.72 (6.48) | 0.574 |
| Inferotemporal | 0.03 (0.01) | **0.019** | -0.13 (0.31) | 0.695 | 0.84 (8.13) | 0.919 |
| Superonasal | 0.02 (0.01) | 0.053 | 0.01 (0.37) | 0.977 | -8.09 (7.79) | 0.315 |
| Inferonasal | 0.03 (0.01) | **0.034** | 0.23 (0.26) | 0.402 | -10.70 (5.85) | 0.092 |
| Nasal Upper | 3.7e-3 (8.3e-3) | 0.666 | -0.21 (0.24) | 0.411 | -5.33 (5.07) | 0.310 |
| Nasal Lower | 2.4e-3 (0.01) | 0.685 | 0.03 (0.24) | 0.913 | 0.44 (5.18) | 0.934 |

Supplemental Table 4: Correlations of RPC vessel density and RNFL thickness with mean sensitivity, TDV, and PSD in the corresponding optic nerve head sector for glaucoma suspect subjects

| Optic Nerve Head sector | R Pearson correlation with mean sensitivity (1/Lambert) | | R Pearson correlation with TDV (1/Lambert) | | R Pearson correlation with PSD (1/Lambert) | |
| --- | --- | --- | --- | --- | --- | --- |
|  | RPC vessel density (95% CI) | RNFL thickness (95% CI) | RPC vessel density (95% CI) | RNFL thickness (95% CI) | RPC vessel density (95% CI) | RNFL thickness (95% CI) |
| Temporal Upper | 0.19 (-0.24, 0.55) | -0.12 (-0.59, 0.38) | 0.01 (-0.41, 0.34) | -0.20 (-0.57, 0.26) | -0.23 (-0.49, 0.03) | -0.42 (-0.64, -0.15) |
| Temporal Lower | 0.17 (-0.36, 0.56) | -0.01 (-0.43, 0.41) | 0.04 (-0.40, 0.39) | -0.01 (-0.46, 0.47) | -0.28 (-0.61, 0.06) | -0.33 (-0.69, 0.23) |
| Superotemporal | 0.19 (-0.13, 0.53) | -0.05 (-0.46, 0.35) | 0.06 (-0.30, 0.43) | -0.09 (-0.47, 0.30) | 0.00 (-0.36, 0.38) | -0.03 (-0.30, 0.34) |
| Inferotemporal | 0.30 (-0.02, 0.53) | 0.31 (-0.19, 0.70) | 0.15 (-0.11, 0.42) | 0.26 (-0.21, 0.63) | 0.24 (0.01, 0.54) | 0.44 (0.13, 0.71) |
| Superonasal | 0.22 (-0.03, 0.50) | 0.01 (-0.41, 0.41) | 0.16 (-0.07, 0.42) | -0.01 (-0.37, 0.38) | 0.07(-0.22, 0.35) | 0.04 (-0.32, 0.38) |
| Inferonasal | 0.33 (-0.05, 0.64) | 0.21 (-0.23, 0.52) | 0.11 (-0.33, 0.50) | 0.31 (-0.02, 0.57) | 0.07 (-0.29, 0.42) | 0.10 (-0.23, 0.46) |
| Nasal Upper | 0.23 (-0.20, 0.60) | 0.08 (-0.42, 0.53) | 0.21 (-0.26, 0.59) | 0.13 (-0.26, 0.48) | 0.26 (-0.16, 0.61) | 0.06 (-0.36, 0.46) |
| Nasal Lower | 0.20 (-0.03, 0.46) | 0.14 (-0.24, 0.51) | 0.19 (-0.07, 0.47) | 0.17 (-0.15, 0.48) | 0.10 (-0.26, 0.41) | 0.19 (-0.15, 0.49) |

Supplemental Table 5: Effect of mean sensitivity measured in decibels on RPC vessel density or RNFL thickness adjusting for patient demographics in glaucoma suspect subjects

| Optic Nerve Head sector | Estimate for RPC (Vessel Density %) | | | | | |
| --- | --- | --- | --- | --- | --- | --- |
|  | Mean Sensitivity (dB, SE) | P Value | Age (years, SE) | P Value | Male Gender (SE) | P Value |
| Temporal Upper | -0.07 (0.31) | 0.829 | -0.23 (0.07) | 0.013 | 0.29 (1.67) | 0.867 |
| Temporal Lower | 0.08 (0.53) | 0.884 | -0.19 (0.09) | 0.077 | 1.85 (2.51) | 0.493 |
| Superotemporal | 0.21 (0.38) | 0.628 | -0.08 (0.08) | 0.361 | 0.51 (2.55) | 0.850 |
| Inferotemporal | 0.01 (0.49) | 0.991 | -0.22 (0.16) | 0.197 | -4.86 (5.70) | 0.43 |
| Superonasal | 0.36 (0.27) | 0.232 | 0.01 (0.12) | 0.931 | 2.44 (3.03) | 0.456 |
| Inferonasal | 0.02 (0.47) | 0.962 | -0.20 (0.08 | **0.040** | 1.62 (2.71) | 0.572 |
| Nasal Upper | 0.41 (0.42) | 0.344 | -0.08 (0.09) | 0.437 | 0.154 (2.90) | 0.96 |
| Nasal Lower | 0.45 (0.51) | 0.402 | -0.01 (0.12) | 0.908 | 3.08 (3.05) | 0.354 |
|  | Estimate for RNFL (um) | | | | | |
| Temporal Upper | -1.51 (1.32) | 0.319 | -0.21 (0.24) | 0.42 | -0.89 (7.61) | 0.91 |
| Temporal Lower | -0.19 (1.16) | 0.876 | -0.07 (0.16) | 0.673 | 1.00 (3.30) | 0.774 |
| Superotemporal | -0.82 (1.20) | 0.54 | -0.06 (0.39) | 0.877 | 13.92 (5.87) | 0.060 |
| Inferotemporal | 2.42 (1.87) | 0.251 | -0.06 (0.32) | 0.860 | -8.27 (7.80) | 0.333 |
| Superonasal | 0.44 (1.16) | 0.720 | 0.12 (0.40) | 0.769 | 17.05 (11.07) | 0.182 |
| Inferonasal | 1.80 (1.86) | 0.366 | 0.06 (0.50) | 0.901 | 22.57 (11.88) | 0.109 |
| Nasal Upper | 1.31 (0.71) | 0.094 | 0.27 (0.26) | 0.344 | 15.67 (7.98) | 0.099 |
| Nasal Lower | 1.82 (1.32) | 0.202 | 0.24 (0.24) | 0.347 | 11.60 (7.58) | 0.179 |

Supplemental Table 6: Effect of mean sensitivity measured in 1/Lambert on RPC vessel density or RNFL thickness adjusting for patient demographics in glaucoma suspect subjects

| Optic Nerve Head sector | Estimate for RPC (Vessel Density %) | | | | | |
| --- | --- | --- | --- | --- | --- | --- |
|  | Mean Sensitivity (1/L, SE) | P Value | Age (years, SE) | P Value | Male Gender (SE) | P Value |
| Temporal Upper | -9.1e-4 (1.3e-3) | 0.512 | -0.25 (0.07) | **0.015** | 0.30 (1.59) | 0.856 |
| Temporal Lower | -1.2e-4 (2.0e-3) | 0.952 | -0.20 (0.09) | 0.064 | 1.75 (2.45) | 0.504 |
| Superotemporal | 9.1e-4 (2.5e-3) | 0.727 | -0.08 (0.09) | 0.403 | 0.29 (2.50) | 0.911 |
| Inferotemporal | 2.3e-4 (2.7e-3) | 0.936 | -0.22 (0.15) | 0.182 | -4.85 (5.57) | 0.419 |
| Superonasal | 3.9e-3 (2.6e-3) | 0.182 | 0.03 (0.13) | 0.805 | 2.52 (2.95) | 0.428 |
| Inferonasal | -4.4e-4 (0.01) | 0.934 | -0.20 (0.09) | **0.041** | 1.56 (2.77) | 0.596 |
| Nasal Upper | 3.5e-4 (2.2e-3) | 0.879 | -0.13 (0.10) | 0.253 | -0.193 (3.21) | 0.954 |
| Nasal Lower | 9.5e-4 (2.7e-3) | 0.741 | -0.05 (0.11) | 0.678 | 2.97 (3.12) | 0.381 |
|  | Estimate for RNFL (um) | | | | | |
| Temporal Upper | -4.4e-3 (5.1e-3) | 0.424 | -0.20 (0.25) | 0.447 | -0.22 (7.52) | 0.978 |
| Temporal Lower | -4.4e-4 (4.6e-3) | 0.926 | -0.07 (0.16) | 0.690 | 1.07 (3.31) | 0.758 |
| Superotemporal | -0.01 (0.01) | 0.419 | -0.09 (0.40) | 0.828 | 15.04 (5.47) | **0.036** |
| Inferotemporal | 0.02 (0.01) | 0.217 | 0.01 (0.33) | 0.980 | -8.43 (7.88) | 0.328 |
| Superonasal | 0.01 (0.01) | 0.585 | 0.16 (0.41) | 0.706 | 17.30 (10.79) | 0.164 |
| Inferonasal | 0.03 (0.02) | 0.161 | 0.21 (0.48) | 0.671 | 24.71 (12.14) | 0.091 |
| Nasal Upper | 3.5e-3 (0.01) | 0.514 | 0.152 (0.25) | 0.571 | 14.73 (8.54) | 0.137 |
| Nasal Lower | 4.9e-3 (0.01) | 0.617 | 0.12 (0.26) | 0.659 | 11.10 (7.92) | 0.214 |
